# Acquired Hemophilia A with a Rare Presentation of Acute Subdural Hematoma

**DOI:** 10.1155/2015/543927

**Published:** 2015-09-29

**Authors:** Yoshihide Sehara, Yuka Hayashi, Jun Mimuro

**Affiliations:** ^1^Department of Neurology, Ishibashi General Hospital, Shimotsuke 329-0596, Japan; ^2^Department of Neurology, Jichi Medical University, Shimotsuke 329-0498, Japan; ^3^Department of Hematology, Ishibashi General Hospital, Shimotsuke 329-0596, Japan

## Abstract

An 80-year-old man was admitted for acute subdural hematoma caused by a mild brain injury. His coagulation test showed an isolated prolongation of activated partial thromboplastin time (aPTT). Though the subdural hematoma did not progress, oozing bleed from the wound of tracheostomy continued. Failure of correction on aPTT mixing test supported the presence of an inhibitor to a coagulation factor. Once the diagnosis of acquired hemophilia A (AHA) was made, steroid therapy was performed, which leads him to complete remission of AHA. Isolated prolongation of aPTT can be the key to diagnose a rare coagulopathy, such as AHA.

## 1. Introduction

Acquired hemophilia A (AHA) is a rare disorder (approximately 1 in 1 million persons per year) caused by an autoantibody to factor VIII [1]. Well-recognized risk factors for AHA are malignancy, autoimmune diseases, and pregnancy; however, approximately 50% of cases are idiopathic [2]. In recent studies, the overall mortality rate has been reported to be 31% and 33% [2, 3]. Here, we describe a patient with acquired hemophilia A presenting with acute subdural hematoma caused by a mild traumatic brain injury, which was successfully treated with steroid therapy.

## 2. Case Presentation

**Figure 1:**
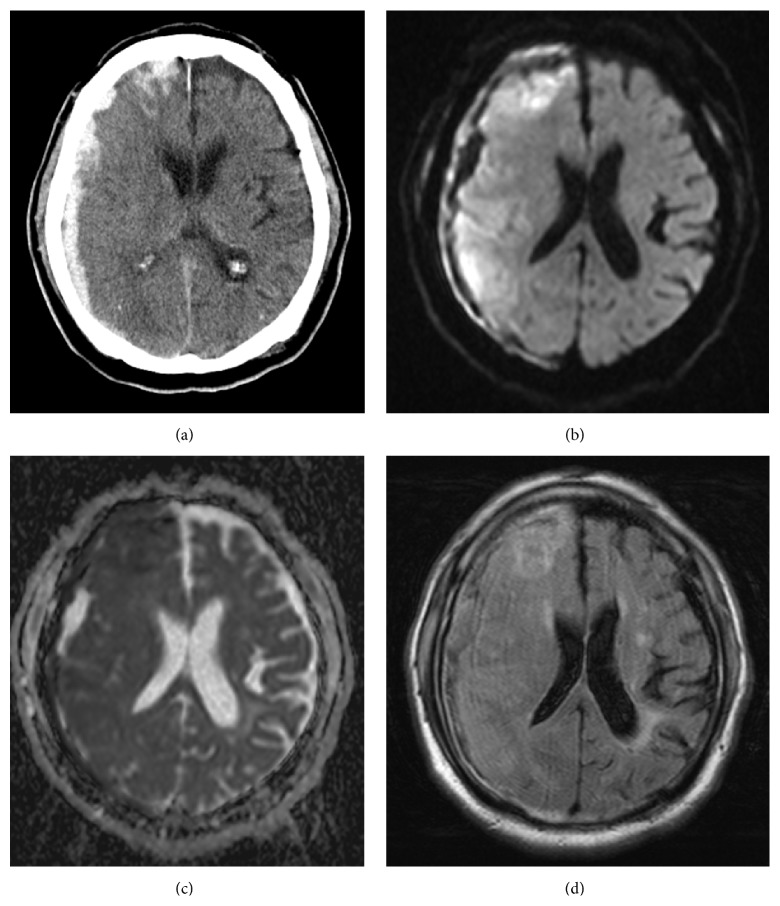
Computed tomography (CT) and magnetic resonance image (MRI) on admission. Brain CT shows a right hemispheric subdural hematoma (a). Diffusion-weighted image of brain MRI shows high signal intensity in frontal and temporal lobes (b). Apparent diffusion coefficient map is low (c) and fluid attenuated inversion recovery shows high signal intensity (d) in the corresponding areas.

An 80-year-old man was admitted to an ambulance center for left hemiplegia followed by a fall from a bed (30 cm: height) before he came to our hospital. He had a past history of cerebral infarction and myocardial stroke, which showed no sequelae. He had been taking antiplatelet therapy until admission. The computed tomography (CT) scan on admission disclosed a right hemispheric subdural hematoma and the magnetic resonance image (MRI) disclosed brain contusion of front and temporal lobes (Figure 1). He had status epilepticus on the 2nd day of admission and was intubated. He had tracheostomy due to prolonged consciousness disturbance on the 13th day of admission and oozing bleed from the wound of tracheostomy continued. Because the activated partial thromboplastin time (aPTT) was prolonged (104.2 sec) and the anemia (68 g/L: hemoglobin) was progressed, fresh frozen plasma and red blood cell concentration were transfused on the 25th and 26th day of admission. After a conservative treatment of acute subdural hematoma and brain contusion, he was transferred to our hospital on the 26th day of admission.

At presentation, he was alert, his blood pressure was 148/65 mmHg, and his heart rate was 71 beats/min. He was tracheostomized and showed left hemiplegia. His skin had no obvious purpura. The blood count revealed a white blood cell count of 7.2*∗*10^9^/L, hemoglobin of 77 g/L, and platelet of 187*∗*10^9^/L. The coagulation test results showed prothrombin time (PT) of 10.6 sec with an INR of 1.10 and an aPTT of 73.0 sec. He was transfused with fresh frozen plasma one day before the coagulation test, but the aPTT was still prolonged. The nature of aPTT prolongation was studied by mixing the patient's plasma with normal pooled plasma. Failure of correction on aPTT mixing test supported the presence of an inhibitor and promoted further evaluation of clotting factors. Factor VIII (FVIII) activity was decreased to 4% of the normal control, and FVIII inhibitor was detected with a titer of 3 Bethesda units/mL. The antinuclear antibody, rheumatoid factor, lupus anticoagulant (LA), and anti-SSA and anti-SSB antibodies were negative. The chest X-ray and plain CT scan of abdomen showed no evidence of malignancy or infection.

**Figure 2:**
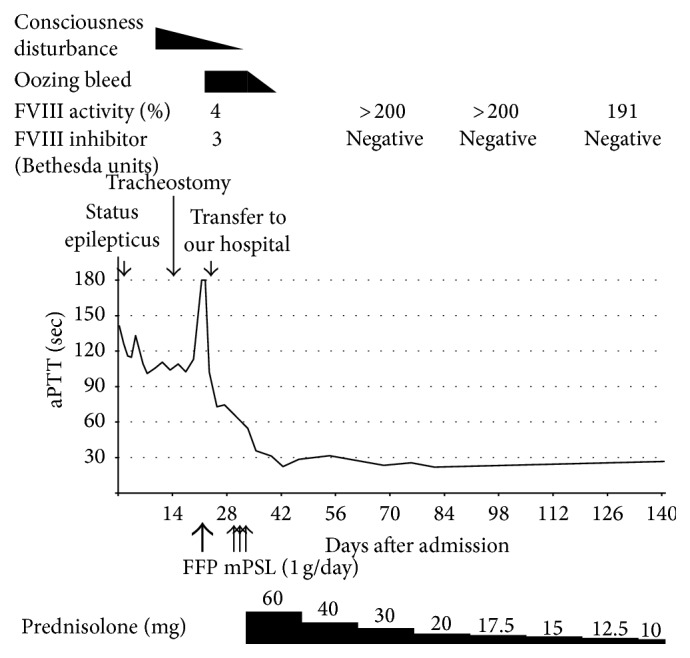
The clinical course. aPTT: activated partial thromboplastin time, FVIII: factor VIII, FFP: fresh frozen plasma, and mPSL: methyl prednisolone.

Once the diagnosis of AHA was made, he was treated with methylprednisolone pulse therapy (3 days, 1 g/day) followed by oral prednisolone therapy (1 mg/kg body weight). Oozing bleed from the tracheostomy stopped and aPTT returned to a normal value of 31.3 sec 10 days after initiation of this therapy. At present, the oral prednisolone is being tapered and FVIII activity has increased to normal value while Bethesda assay now showed undetectable level (Figure 2).

## 3. Discussion

AHA is a rare bleeding disorder characterized by the development of autoantibodies to FVIII. The diagnosis of AHA is most frequently suggested by the clinical evidence of bleeding tendency and confirmed by laboratory investigation. A major characteristic of AHA is the difference in bleeding pattern compared with congenital hemophilia. Bleeding symptoms of AHA may be spontaneous or occur after trauma. Most patients with AHA have hemorrhages into skin, muscles, soft tissues, or mucous membranes. By contrast, haemarthroses, the hallmarks of congenital hemophilia, are uncommon in AHA [4]. The incidence of AHA is estimated to 1 per million per year and increases with age [1]. In recent studies, the overall mortality rate has been reported to be 31% and 33% [2, 3]. Bleeding in AHA is often severe, with reported mortalities of 9–22% in earlier studies and recently 3.3% and 3.5% in European Acquired Haemophilia Registry (EACH2) and Surveillance des Auto antiCorps au cours de l'Hémophilie Acquise (SACHA) registry, respectively [1–3, 5]. In EACH2 registry, AHA was idiopathic in 51.9%; malignancy, autoimmune diseases, or infections were associated with 11.8%, 11.6%, and 2.8% of cases [6]. In this case, his laboratory tests did not reveal these underlying disorders.

In this case, the patient presented acute subdural hematoma after a mild shock. To date, only 5 cases of AHA presenting as an intracranial hemorrhage have been reported. Bonnaud et al. reported a case of spontaneous subdural hematoma with AHA without any history of cranial trauma or fall [7]. They indicated that coagulation screening was usually restricted to presurgical tests in spontaneous subdural hematoma and the diagnosis of underlying coagulopathy was probably underestimated.

Isolated prolongation of aPTT can be seen in patients with deficiency of either factor VIII, IX, or XI, contact factor deficiency (factor XII or prekallikrein deficiency), acquired clotting factor inhibitors (commonly directed against factor VIII), and antiphospholipid antibody syndrome (APS) [4]. In this case, isolated aPTT with normal prothrombin time, normal platelet count, and the mixing test, which was compatible with an inhibitor, were the key to an undiagnosed bleeding disorder. It was important to rule out nonspecific inhibitors (LA or heparin). Sometimes, it is hard to differentiate APS from AHA; however, APS usually shows thrombotic tendency. The diagnosis of AHA was settled by the existence of FVIII inhibitor. His immediate recovery of bleeding tendency and the coagulation test after steroid therapy also supported the diagnosis of AHA.

This patient had been treated with antiplatelet due to old cerebral infarction and coronary heart disease. Prehospital antiplatelet therapy is thought to be associated with significantly increased mortality and unfavorable outcome in traumatic brain injury patients or with higher rates of neurosurgical interventions as well as more episodes of rebleeding [8]. Also, it is shown that the risk of coronary thrombosis after withdrawal of antiplatelet medication is greater than the risk of surgical bleeding if antiplatelet medication is continued [9]. So, because the patient had another problem of coagulopathy, AHA, antiplatelet therapy might be reinitiated immediately after the coagulation test returned to normal.

Because he showed left hemiplegia and was bedridden, the patient had a high risk for venous thromboembolism (VTE). We thought it was necessary to reinitiate the antiplatelet therapy as indicated above, so we performed physiotherapy for his VTE prevention. Generally, dual therapy of antiplatelet and anticoagulant has a high risk for bleeding complication.

It is recommended to immediately initiate antihemorrhagic treatment in patients with AHA and active severe bleeding symptoms, but not all types of bleeding require intervention [4]. Two therapeutic interventions to control bleeding in patients with AHA are available: bypassing agents (recombinant activated FVII and activated prothrombin complex concentrate) and strategies to increase FVIII levels (FVIII concentrate and 1-desamino-8-D-arginine-vasopressin). Bypassing agents are recommended as first-line therapy because of their rapid action and high level of effectiveness [10]. In our case, acute subdural hematoma occurred following a mild brain injury, but it did not enlarge. And oozing bleed from the wound of tracheostomy was seen, but it immediately disappeared after steroid therapy.

Immunosuppressive treatment to eradicate inhibitory autoantibodies to FVIII should be started immediately following the diagnosis. The optimal therapeutic strategy is unknown, but the current recommendations include steroids alone or steroids plus cyclophosphamide [11]. In EACH2, steroids alone have shown a 48% complete remission (CR) rate while steroids and cyclophosphamide showed a 70% with no difference in overall survival [2]. Although the usefulness of steroid pulse therapy has not been reported, we performed steroid pulse therapy (3 days, 1 g/day, methylprednisolone) to expect an earlier and better eradication of FVIII inhibitor. EACH2 showed that the baseline FVIII activity and inhibitor concentration could be used to define a subgroup of patients with better prognosis. A subgroup with a lower inhibitor titer (<20 Bethesda units/mL) and a higher baseline FVIII activity (≥1 IU/dL) was found to have >50% chance of achieving PR with steroids alone by day 21 [12]. In one case, recurrence of spontaneous cerebellar and cerebral hemorrhage with FVIII activity at 13% and FVIII inhibitor titer 1.7 Bethesda units/mL was expired [13]. In the cases of AHA with intracranial hemorrhage, the treatment might be more difficult than other typical cases.

Currently, there is no rationale for the use of intravenous immunoglobulin alone or in combination with steroids. Recently, rituximab has emerged as a promising new agent for eradication of inhibitors in patients with AHA. The recommendations indicated rituximab, when used in conjunction with steroid or cytotoxic therapy, as a safe and effective first- or second-line therapy [4].

Our case successfully leads to CR using steroids alone, but steroids plus cyclophosphamide or rituximab-based regimen might be considered in case of relapse.
